# Susceptibility of *Phthorimaea absoluta* (Meyrick) (Lepidoptera: Gelechiidae) to novel and established insecticides in Brazil: resistance survey, baseline, and implications for management

**DOI:** 10.1002/ps.70669

**Published:** 2026-02-22

**Authors:** Vitor Quintela, Teófilo P Langa, José VCR Dantas, Marcos de Oliveira, Herbert AA Siqueira

**Affiliations:** ^1^ Universidade Federal Rural de Pernambuco Recife Brazil; ^2^ Universidade Eduardo Mondlane Inhambane Mozambique; ^3^ Universidade Eduardo Mondlane Centro de Excelência em Sistemas Agroalimentares e Nutrição (CE‐AFSN) Maputo City Mozambique

**Keywords:** *Tuta absoluta*, insecticide resistance, isocycloseram, tolfenpyrad, diagnostic concentration, synergism

## Abstract

**BACKGROUND:**

*Phthorimaea (=Tuta) absoluta* (Meyrick) (Lepidoptera: Gelechiidae) is a major tomato pest worldwide, and its management is increasingly threatened by rapid resistance evolution. The recent introduction of isocycloseram and tolfenpyrad—two insecticides with novel modes of action—offers new control options, but their sustainability depends on establishing susceptibility baselines and enabling early resistance detection.

**RESULTS:**

Bioassays with 17 Brazilian populations revealed high susceptibility to isocycloseram (LC_50_ = 0.00047–0.0143 mg L^−1^) and to tolfenpyrad (LC_50_ = 0.48–6.86 mg L^−1^), with resistance ratios up to 30‐ and 14‐fold, respectively. Diagnostic concentrations of 47 mg L^−1^ (tolfenpyrad) and 0.3 mg L^−1^ (isocycloseram) were derived from LC_99_ values from set of curves. Monitoring showed that abamectin, fipronil, and indoxacarb displayed widespread resistance (f ≥ 1%). Synergism assays implicated esterases, GSTs, and cytochrome P450s in detoxification, especially for abamectin and partially for isocycloseram. Significant correlations between isocycloseram and tolfenpyrad (r_p_ = 0.732) and between fipronil and indoxacarb (r_p_ = 0.807) suggest shared metabolic pathways or concurrent susceptibility patterns.

**CONCLUSION:**

*P. absoluta* populations are susceptible to isocycloseram and tolfenpyrad, but early tolerance shifts signal emerging adaptive responses that merit close monitoring. The enzymatic associations observed highlight the central role of metabolic detoxification in shaping susceptibility and potential cross‐resistance. Overall, the new chemistries remain useful options for *P. absoluta* control, but their long‐term effectiveness depends on early identification of susceptibility shifts and incorporating metabolic insights into resistance management strategies. © 2026 The Author(s). *Pest Management Science* published by John Wiley & Sons Ltd on behalf of Society of Chemical Industry.

## Graphical abstract

Baseline susceptibility of *Phthorimaea absoluta* to novel insecticides isocycloseram and tolfenpyrad was established in Brazil. Diagnostic doses were proposed for early resistance detection. Early tolfenpyrad tolerance and abamectin‐driven cross‐tolerance to isocycloseram *via* oxidases highlight cross‐resistance risks, emphasizing the need for routine monitoring within integrated resistance management strategies.

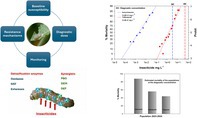

## 1 INTRODUCTION

Understanding why we must monitor *Phthorimaea* (=*Tuta*) *absoluta* (Meyrick) (Lepidoptera: Gelechiidae) precedes the question of how. This pest, known as the South American tomato pinworm, poses a persistent global threat to solanaceous crops—particularly tomato (*Solanum lycopersicum*)—through its rapid reproductive cycle, cryptic feeding habits, and extraordinary capacity to evolve resistance across diverse insecticide classes. Originating from South America, *P. absoluta* has, within two decades, spread across Europe, Africa, and Asia, becoming a model for the global challenge of resistance management.^1^, ^2^, ^3^, ^4^ In Brazil, where tomato production heavily depends on chemical control,^2^ this pest remains a central obstacle. Historically, organophosphates, pyrethroids, nereistoxin derivatives, chitin‐synthesis inhibitors, avermectins, spinosyns, and diamides were the main control tools. Yet the intense application frequency, coupled with rapid generational turnover, has led to widespread resistance, eroding product efficacy^5^ and accelerating selection pressure against newer compounds.

Although several insecticide classes are available to support rotation among different modes of action (MoA), multiple‐resistance profiles have already emerged in Brazilian populations—spanning pyrethroids, diamides, and avermectins.^6^ This indicates that rotation schemes, in practice, have not been effectively applied. Field reports confirm reduced efficacy of benzoylureas, spinosyns, and diamides,^7^, ^8^, ^9^ and the indiscriminate alternation of unrelated MoAs has likely selected for populations carrying resistance alleles to several insecticide classes simultaneously.

Recognizing this, recent initiatives in integrated resistance management (IRM) have stressed the why: delaying resistance evolution and restoring sustainable control depend on diversifying chemistries and understanding susceptibility baselines. Among the novel insecticides introduced in Brazil for *P. absoluta* control are tolfenpyrad, a mitochondrial complex I electron transport inhibitor (IRAC Group 21A),^10^ and isocycloseram, an isoxazoline targeting GABA‐gated chloride channels (IRAC Group 30).^11^ These molecules provide new alternatives to older compounds such as abamectin (a glutamate‐gated chloride channel agonist, IRAC Group 6),^12^, ^13^ indoxacarb (a voltage‐gated sodium channel blocker, IRAC Group 22A),^14^ and fipronil (a phenylpyrazole GABA antagonist, IRAC Group 2B).^15^

However, their success hinges on how we deploy them, grounded in a clear understanding of baseline susceptibility, potential cross‐resistance with existing chemistries, and the enzymatic mechanisms that drive detoxification. Establishing such baselines is a preventive act, the foundation for early resistance detection and rational product use, as emphasized in IRAC guidelines.^16^, ^17^ Beyond the quantitative assessment of susceptibility, synergism assays offer what we need to interpret the biology behind resistance. By pairing insecticides with enzyme inhibitors, these assays identify which detoxification systems—cytochrome P450s, esterases, or glutathione *S*‐transferases—mediate tolerance.^18^ This metabolic dimension sharpens our understanding of cross‐resistance and reveals potential vulnerabilities that can be exploited within IRM programs.

In this study, we investigated the susceptibility of *P*. *absoluta* to two newly introduced insecticides across field and laboratory populations collected from major tomato‐producing regions in Brazil. Our primary goal was to establish robust baseline data to support future monitoring and resistance management programs. Specifically, we aimed to (i) estimate the concentration‐mortality for both compounds across geographically distinct populations, (ii) define diagnostic concentrations capable of detecting low‐frequency resistance alleles in field monitoring, (iii) evaluate potential cross‐resistance patterns between isocycloseram, and insecticides with distinct modes of action acting on chloride ion channel, and (iv) explore the metabolic basis of tolerance using synergism bioassays with enzyme inhibitors. We hypothesized that despite the novelty of both compounds, variations in susceptibility among Brazilian populations would reveal early signs of tolerance possibly mediated by detoxification enzymes previously selected by older chemistries. By integrating concentration‐response, diagnostic, and synergism assays, this study provides an essential foundation for understanding how emerging resistance to new insecticides may evolve in *P. absoluta* and informs the design of proactive IRM strategies in Brazil.

## 2 MATERIALS AND METHODS

### 2.1 Insect collection and rearing

**Figure 1:**
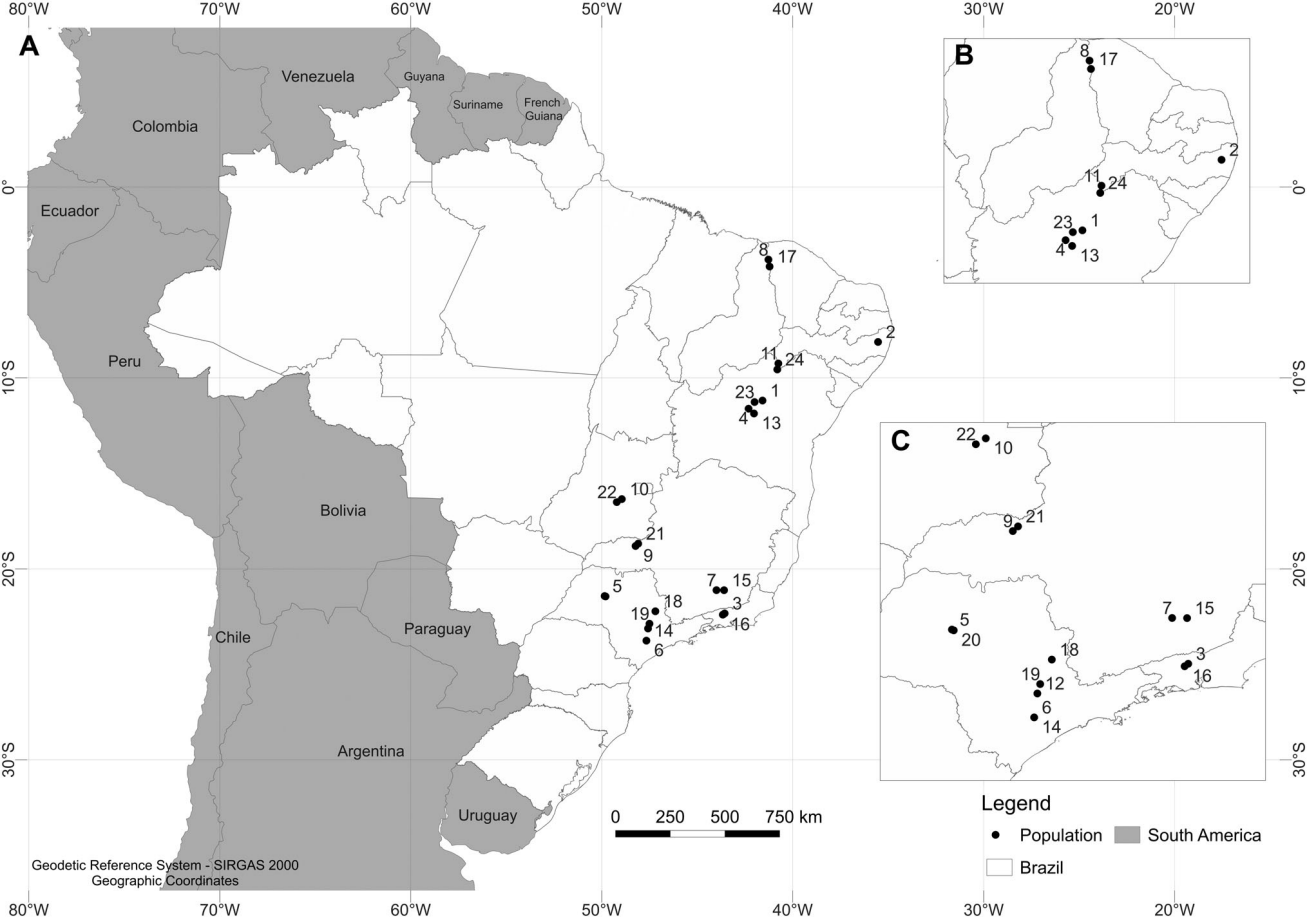
Sampling sites distribution of *P. absoluta* populations in Brazil.

Populations of *P. absoluta* (Meyrick) were collected from infested tomato (*S. lycopersicum* L.) fields across the Southeast, Midwest, and Northeast regions of Brazil (Table 1, Fig. 1). Field‐collected larvae were transported to the laboratory and maintained in rearing cages (45 × 45 × 45 cm). Upon adult emergence, insects were transferred to oviposition cages (30 × 30 × 30 cm) and provided with a 10% honey solution supplemented with yeast as a protein source. Rearing was conducted under controlled environmental conditions: 25 ± 1 °C, 65 ± 5% relative humidity (RH), and a 12:12 h light: dark photoperiod. Tomato leaves from the cultivar ‘Santa Clara’ (IC 5500) were used both for oviposition and as larval food.

**Table 1:** Population ID, collecting site, and time of collection sampling of *Phthorimaea absoluta*

Population	Map # ref.	Municipality/State	Coordinates	Collection date
JDR1‐Sus	1	João Dourado/BA	−11.19, −41.38	Aug. 2014^a^
GVT‐Aba	2	Gravatá/PE	−08.12, −35.33	Dec. 2020^a^
PTY‐Espin	3	Paty do Alferes/RJ	−22.327859, −43.362572	Nov. 2022
LGD‐Clora	4	Lagoa Grande/BA	−11.618525, −42.110752	Jun. 2019^a^
LIN‐Sus	5	Sabino (Lins)/SP	−21.417983, −49.6468660	Jul. 2023
PIE‐2023	6	Piedade/SP	−23.7551655, −47.4596400	Jul. 2023
BAR‐2023	7	Barbacena/MG	−21.109566, −43.791125	Nov. 2023
TNG‐2023	8	Tianguá/CE	−03.8035431, −41.069854	Jun. 2023
ARA‐2023	9	Araguari/MG	−18.79613, −48.02722	Dec. 2023
ANA‐2023	10	Anápolis/GO	−16.33787, −48.74729	Dec. 2022
PET‐2023	11	Petrolina/PE	−09.247994, −40.550358	Sept. 2023
EFA‐2023	12	Elias Fausto/SP	−23.11885, −47.37479	Jun. 2023
BAL‐2023	13	Barro Alto/BA	−11.869068, −41.826384	Sept. 2023
PIE‐2024	14	Piedade/SP	−23.75516, −47.45964	Mar. 2024
BAR‐2024	15	Barbacena/MG	−21.11220, −43.39367	May 2024
PTY‐2024	16	Paty do Alferes/RJ	−22.39450, −43.45840	May 2024
CNB‐2024	17	Tianguá/CE	−04.167159, −41.008433	May 2024
AGU‐2024	18	Aguaí/SP	−22.21654, −46.99021	Aug. 2024
SUM‐2024	19	Sumaré/SP	−22.86829, −47.29963	Aug. 2024
LIN‐2024	20	Sabino (Lins)/SP	−21.43816, −49.59932	Aug. 2024
ARA‐2024	21	Araguari/MG	−18.67173, −47.88870	Sept.2024
GOI‐2024	22	Goianápolis/GO	−16.49353, −49.01070	Sept. 2024
IRE‐2024	23	Irecê/BA	−11.26876, −41.79560	Oct.2024
JUA‐2024	24	Juazeiro/BA	−09.56126, −40.60477	Oct. 2024

^a^ Laboratory population.

### 2.2 Insecticides and chemicals

The insecticides used in bioassays were as follows: isocycloseram (Joiner® 200 g L^−1^, emulsifiable concentrate, Syngenta SA, São Paulo, Brazil), tolfenpyrad (Ohkami® 100 g L^−1^, oil‐in‐water emulsion, Nichino do Brasil Agroquímicos Ltda, Barueri, São Paulo), abamectin (Vertimec® 84 g L^−1^, emulsifiable concentrate, Syngenta SA, São Paulo, Brazil), fipronil (Regent® 80% m/m, granule, BASF SA, São Paulo, Brazil), and indoxacarb (Avatar® 150 g L^−1^, emulsifiable concentrate, FMC LTDA, São Paulo, Brazil). Metabolic synergists included piperonyl butoxide (PBO, 90%), *S, S, S*‐tributyl phosphorotrithioate (DEF, 98%), and diethyl maleate (DEM, 97%), all purchased from Sigma‐Aldrich (Milwaukee, WI, USA).

### 2.3 Leaf‐dip bioassay

Dose–response bioassays were conducted in 2024 on 11 field populations and six laboratory‐maintained populations (JDR1‐Sus, GVT‐Aba, PTY‐2023, LIN‐2023, LGD‐Clora and IRE‐2023) using the novel insecticides isocycloseram (IRAC MoA 30) and tolfenpyrad (IRAC MoA 21A). The standard leaf‐dip method was employed according with Quintela (2025) adapted from IRAC.^6^, ^19^ Tomato leaflets were immersed in insecticide solutions containing 0.01% of the adjuvant Break‐Thru® (Evonik Degussa, Brazil), air‐dried, and placed in Petri dishes lined with moistened filter paper. Ten second‐instar larvae were placed per dish, sealed and incubated at 25 ± 1 °C, 65 ± 5% RH, and a 12:12 h photoperiod. Mortality was assessed at 48 h for isocycloseram and at 96 h for tolfenpyrad. Larvae were considered dead if they did not respond to gentle prodding with a soft brush.

### 2.4 Diagnostic dose monitoring and control failure likelihood (CFL)

The diagnostic dose screening with abamectin (5 mg L^−1^),^6^ fipronil (10 mg L^−1^),^20^ and indoxacarb (10 mg L^−1^)^5^, ^21^ was conducted on 22 populations collected during 2023 and 2024. The diagnostic dose assays were then conducted using the same procedure as the leaf‐dip bioassay, with 10 replicates of 10 individuals per plate. The susceptible reference colony (JDR1‐Sus), previously used for abamectin study,^6^ was included in all assays for comparative purposes. Applications of commercial label doses—18 mg L^−1^ for abamectin and 48 mg L^−1^ for indoxacarb—were also tested to find control failure likelihood.

### 2.5 Cross‐resistance to chloride channel modulators

Given the MoA of isocycloseram as a GABA‐gated chloride channel modulator, potential cross‐resistance was investigated with abamectin and fipronil. Concentration‐mortality curves were generated using both abamectin‐resistant and ‐susceptible colonies. Parameters of probit regression models were estimated and compared using lethal ratio tests to determine cross‐resistance patterns.

### 2.6 Synergism bioassays

Preliminary assays using 10‐fold dilution of each synergist (varying from 0.001 to 10 μg uL^−1^, in acetone) were performed to determine appropriate synergist concentrations that did not cause mortality or visible behavioral effects in larvae. Based on these trials, concentrations were selected that allowed normal survival while still effectively inhibiting their respective enzyme systems. Second‐instar larvae from resistant and susceptible colonies were then exposed to abamectin or isocycloseram, either alone or in combination with the metabolic inhibitors piperonyl butoxide (PBO, cytochrome P450 monooxygenase inhibitor), S,S,S‐tributyl phosphorotrithioate (DEF, esterase inhibitor), and diethyl maleate (DEM, glutathione S‐transferase inhibitor). Synergists were dissolved in acetone at 1 μg μL^−1^, and each larva was topically treated with 2 μL using a Hamilton microsyringe. Approximately 30 min after treatment, larvae were transferred to tomato leaflets previously treated with insecticide. Leaf surfaces were allowed to dry completely before exposure. Control treatments included solvent‐only applications for both synergists and insecticides.

### 2.7 Data analysis

Probit analyses were performed using POLO Plus software to estimate LC₅₀, LC_90_ and LC₉₉ values, slope parameters, and 95% confidence intervals. Mortality was corrected using Abbott's formula.^22^ Resistance ratios (RR) were calculated relative to the susceptible strain, with significance inferred when the 95% confidence interval of the RR excluded 1.0^23^ Survival at diagnostic concentrations was evaluated using one‐sided Z‐tests with continuity correction^24^ at the 5% significance level, run within R environment (v4.4.3).^25^ A critical Z‐value of 1.645 was used to determine if observed survival exceeded the expected 1% frequency threshold, following Roush and Miller (1986)^26^ resistance classification criteria. Pearson correlation coefficients were calculated using R (v4.4.3) and the *corrplot* package^27^ to evaluate associations between LC₅₀ values and percentage mortality across insecticides with correction of false discovery rate (FDR).^28^ The control failure likelihood (CFL) was calculated using the formula CFL = 100 ‐ [observed mortality (%) × 100]/expected mortality (%),^29^ with 80% assumed as the field efficacy threshold.

## 3 RESULTS

### 3.1 Baseline susceptibility of *P. absoluta* to isocycloseram and tolfenpyrad

Lethal concentration values (LC₅₀) for isocycloseram and tolfenpyrad were estimated for 11 field populations of *P. absoluta* collected in seven Brazilian states throughout 2024, along with six laboratory populations (Fig. 1 and Table 1). For tolfenpyrad, the PTY‐2024 population was the most susceptible (LC₅₀ = 0.48 mg L^−1^). Low variability was observed among PIE‐2024, IRE‐2023, PTY‐2023, BAR‐2024, LIN‐2024, JUA‐2024, SUM‐2024, IRE‐2024, AGU‐2024, LGD‐Clora, JDR1‐Sus and LIN‐2023 (LC₅₀ = 1.04–1.85 mg L^−1^). Moderate tolerance (LC₅₀ = 2.28–3.68 mg L^−1^) was found in CNB‐2024, ARA‐2024, and GVT‐Aba, while GOI‐2024 showed the highest LC₅₀ (6.86 mg L^−1^) (Table 2 and Fig. S2). Resistance ratios relative to the most susceptible population (PTY‐2024) ranged from 2.15‐fold in PIE‐2024 to 14.13‐fold in GOI‐2024 (Table 2).

**Table 2:** Susceptibility of first instar larvae of *Phthorimaea absoluta* to tolfenpyrad

Population (G)	*N* ^a^	Slope ± SE^b^	LC50 (95% CI)^c^	LC99 (95% CI)^c^	*ꭓ* ^2^ (Prob., DF)^d^	RR_50_ (95% CI)^e^
PTY‐2024 (F1)	320	0.79 ± 0.11	0.48 (0.23–0.78)	411 (110.91–4298.5)	1.76 (0.94, 6)	–
PIE‐2024 (F1)	320	1.41 ± 0.15	1.04 (0.73–1.41)	45.83 (24.32–117.67)	2.27 (0.89, 6)	2.15 (1.11–4.15)*
IRE‐2023 (F8)	240	1.31 ± 0.16	1.05 (0.71–1.47)	61.78 (28.35–214.63)	1.23 (0.97, 6)	2.17 (1.10–4.27)*
PTY‐2023 (F18)	320	2.22 ± 0.31	1.07 (0.73–1.43)	11.93 (7.54–25.42)	2.76 (0.83, 6)	2.22 (1.15–4.28)*
BAR‐2024 (F1)	322	1.25 ± 0.13	1.20 (0.88–1.60)	85.24 (41.42–248.61)	1.83 (0.93, 6)	2.48 (1.30–4.74)*
LIN‐2024 (F1)	323	2.45 ± 0.30	1.27 (0.96–1.60)	11.26 (7.49–21.30)	1.87 (0.93, 6)	2.62 (1.40–4.90)*
JUA‐2024 (F2)	323	1.64 ± 0.15	1.29 (0.88–1.85)	33.76 (15.87–122.44)	8.60 (0.19, 6)	2.66 (1.43–4.96)*
SUM‐2024 (F1)	320	1.51 ± 0.14	1.33 (0.87–1.99)	46.21 (19.78–206.35)	9.31 (0.18: 6)	2.75 (1.47–5.16)*
IRE‐2024 (F1)	318	1.36 ± 0.17	1.42 (0.70–2.33)	72.34 (29.04–418.54)	7.13 (0.30, 6)	2.92 (1.44–5.92)*
AGU‐2024 (F1)	319	1.68 ± 0.18	1.51 (1.11–1.99)	36.76 (21.36–81.95)	4.23 (0.64, 6)	3.12 (1.64–5.93)*
LGD‐Clora (F25)	320	1.14 ± 0.14	1.61 (1.02–2.32)	173.53 (76.31–635.15)	3.39 (0.75, 6)	3.33 (1.65–6.70)*
JDR1‐Sus (F122)	328	2.40 ± 0.26	1.72 (1.35–2.12)	16.04 (10.76–29.01)	2.48 (0.87, 6)	3.54 (1.91–6.55)*
LIN‐2023 (F18)	320	2.91 ± 0.50	1.85 (1.31–2.33)	11.63 (7.74–25.11)	4.74 (0.57, 6)	3.81 (2.02–7.17)*
CNB‐2024 (F2)	320	1.95 ± 0.40	2.28 (1.04–3.40)	35.19 (19.89–121.50)	5.21 (0.51, 6)	4.70 (2.16–10.19)*
ARA‐2024 (F1)	321	2.02 ± 0.25	2.49 (1.45–3.39)	21.33 (13.30–57.29)	5.29 (0.50, 6)	5.14 (2.58–10.28)*
GVT‐Aba (F36)	320	1.07 ± 0.12	3.68 (2.66–5.29)	523.69 (194.12–2650.20)	4.09 (0.66, 6)	7.59 (3.90–14.79)*
GOI‐2024 (F1)	321	2.33 ± 0.44	6.86 (4.27–9.19)	68.06 (39.97–205.85)	4.69 (0.58, 6)	14.13 (7.22–27.66)*
**Total**	**5382**	**1.52 ± 0.04**	**1.42 (1.14–1.72)**	**47.58 (32.09–78.81)**	**82.65 (0.0, 15)**	—

^a^ Total number of bioassayed insects.

^b^ Slope and standard error.

^c^ Milligrams of tolfenpyrad per liter water and confidential interval at 95%.

^d^ Chi – square (*α* = 0.05), probability and degree of freedom. If the calculated *χ* ^2^ value is greater than the critical value in the table, the null hypothesis is rejected (the model does not fit the data well).

^e^ Resistance ratio: LC50 of each population compared to the most susceptible population using the lethal ratio test (Robertson *et al*. 2017).

* RR50 is significant at 5% probability if the confidence limits does not include the value 1.0. Bold values refer to estimates obteined from the probit analysis based on the pooled dataset of all populations.

The highest susceptibility to isocycloseram was observed in the JUA‐2024 population (LC₅₀ = 0.00047 mg L^−1^), followed by the LGD‐Clora laboratory strain (LC₅₀ = 0.0005 mg L^−1^). Intermediate LC₅₀ values (0.0015–0.0062 mg L^−1^) were recorded for JDR1‐Sus, SUM‐2024, IRE‐2023, CNB‐2024, PTY‐2024, PIE‐2024, LIN‐2023 IRE‐2024, LIN‐2024, PTY‐2024, BAR‐2024, AGU‐2024, GOI‐2024, and ARA‐2024. Higher tolerance level (LC₅₀ = 0.0143 mg L^−1^) was found in GVT‐Aba population. As detailed in (Table 3 and Fig. S1), resistances ratios (RR_50_) varied from 1.06‐fold in LGD‐Clo to 30.19‐fold in the abamectin‐selected population GVT‐Aba.

**Table 3:** Susceptibility of first instar larvae of *Phthorimaea absoluta* to isocycloseram

Population (G)	*N* ^a^	Slope ± SE^b^	LC50 (95% CI)^c^	LC99 (95% CI)^c^	*ꭓ* ^2^ (Prob., DF)^d^	RR_50_ (95% CI)^e^
JUA‐2024 (F2)	280	1.62 ± 0.30	0.00047 (0.0002–0.0007)	0.012 (0.006–0.445)	1.58 (0.90, 5)	—
LGD‐Clora (F25)	320	1.08 ± 0.14	0.0005 (0.0002–0.0007)	0.070 (0.029–0.303)	4.41 (0.62, 6)	1.06 (0.49–2.26)
JDR1‐Sus (F122)	362	1.43 ± 0.12	0.0015 (0.0011–0.0019)	0.062 (0.034–0.144)	3.30 (0.85, 7)	3.15 (1.66–5.97)*
SUM‐2024 (F1)	320	1.54 ± 0.17	0.0017 (0.0009–0.0026)	0.055 (0.253–0.241)	8.83 (0.18, 6)	3.63 (1.88–7.02)*
IRE‐2023 (F8)	240	1.31 ± 0.17	0.0020 (0.0009–0.0033)	0.173 (0.709–0.865)	4.10 (0.66, 6)	4.20 (1.81–9.76)*
CNB‐2024 (F2)	320	0.96 ± 0.12	0.0023 (0.0013–0.0036)	0.625 (0.222–3.476)	2.85 (0.82, 6)	5.01 (2.37–10.60)*
PTY‐2024 (F1)	320	1.18 ± 0.15	0.0025 (0.0015–0.0038)	0.237 (0.106–0.858)	4.58 (0.59, 6)	5.44 (2.61–11.31)*
PIE‐2024 (F1)	400	1.43 ± 0.17	0.0027 (0.0016–0.0040)	0.114 (0.061–0.300)	2.70 (0.95, 8)	5.83 (2.82–12.03)*
LIN‐2023 (F17)	316	1.59 ± 0.20	0.0029 (0.0018–0.0041)	0.084 (0.046–0.218)	5.23 (0.51, 6)	6.22 (3.07–12.61)*
IRE‐2024 (F1)	316	1.58 ± 0.17	0.0030 (0.0017–0.0046)	0.089 (0.042–0.335)	7.61 (0.26, 6)	6.37 (3.24–12.51)*
LIN‐2024 (F1)	320	1.61 ± 0.15	0.0034 (0.0023–0.0048)	0.096 (0.046–0.323)	7.47 (0.27, 6)	7.29 (3.85–13.78)*
PTY‐Esp (F18)	321	1.75 ± 0.23	0.0035 (0.0022–0.0049)	0.075 (0.043–0.182)	4.84 (0.56, 6)	7.46 (3.70–15.02)*
BAR‐2024 (F1)	320	1.20 ± 0.14	0.0037 (0.0024–0.0054)	0.322 (0.145–1.125)	1.65 (0.94, 6)	7.91 (3.89–16.08)*
AGU‐2024 (F1)	319	1.29 ± 0.15	0.0048 (0.0023–0.0082)	0.302 (0.108–2.276)	8.31 (0.21, 6)	10.20 (5.03–20.70)*
GOI‐2024 (F1)	319	1.23 ± 0.16	0.0053 (0.0031–0.0079)	0.412 (0.179–1.629)	4.73 (0.57, 6)	11.15 (5.34–23.28)*
ARA‐2024 (F1)	328	1.38 ± 0.16	0.0062 (0.0041–0.0088)	0.300 (0.148–9.053)	5.73 (0.45, 6)	13.22 (6.63–26.36)*
GVT‐Aba (F36)	342	1.17 ± 0.11	0.0143 (0.0096–0.0214)	1.350 (0.502–6.884)	8.20 (0.31, 7)	30.19 (15.63–58.28)*
Total	**5388**	**1.14 ± 0.03**	**0.029 (0.017–0.066)**	**0.239 (0.099–1.047)**	**294.7 (0.0, 17)**	—

^a^ Total number of bioassayed insects.

^b^ Slope and standard error.

^c^ Milligrams of isocycloseram per liter water and confidential interval at 95%.

^d^ Chi – square (α = 0.05), probability and degree of freedom. If the calculated χ^2^ value is greater than the critical value in the table, the null hypothesis is rejected (the model does not fit the data well).

^e^ Resistance ratio: LC50 of each population compared to the most susceptible population using the lethal ratio test (Robertson *et al*. 2017).

* RR50 is significant at 5% probability if the confidence limits does not include the value 1.0. Bold values refer to estimates obteined from the probit analysis based on the pooled dataset of all populations.

We propose diagnostic doses of 0.3 mg L^−1^ for isocycloseram and 47 mg L^−1^ for tolfenpyrad, defined from LC₉₉ estimates derived from pooled laboratory and field population data, thereby ensuring that natural variability in susceptibility is captured.

### 3.2 Diagnostic dose monitoring and control failure likelihood

**Figure 2:**
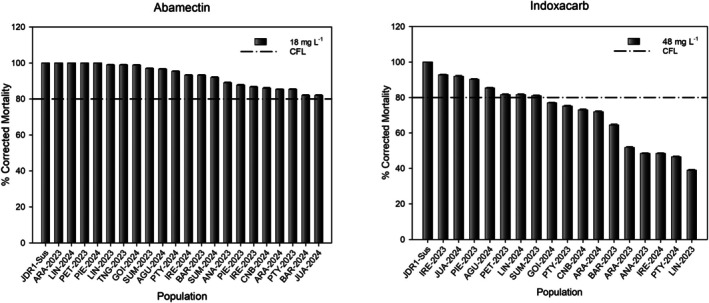
Two‐year survey (2023–2024) of susceptibility of *P. absoluta* to abamectin and indoxacarb using label concentrations (control failure likelihood).

Field populations collected during 2023 and 2024 were evaluated using diagnostic doses of abamectin, fipronil, and indoxacarb. Most of these populations showed resistance frequencies at or above the 1% threshold (f ≥ 1%), indicating that resistance to these insecticides is already widespread (Table 4). We further evaluated the label doses of abamectin (18 mg L^−1^) and indoxacarb (48 mg L^−1^), as these are the most extensively used and registered insecticides for *P. absoluta* in Brazil. Assuming a minimum expected efficacy of 80% mortality, abamectin exhibited low control failure likelihood (CFL) across all tested populations. In contrast, indoxacarb showed high CFL in 10 of the tested populations (Fig. 2).

**Table 4:** Susceptibility of field *Phthorimaea absoluta* populations to diagnostic doses using one‐sided Z‐tests

	Abamectin 5 mg L^−1^				Fipronil 10 mg L^−1^				Indoxacarb 10 mg L^−1^			
Population	*N* ^a^	Mort.	Resist.	Z Score	*N*	Mort.	Resist.	Z Score	*N*	Mort.	Resist.	Z Score
JDR1‐Sus	100	100	0.0	−1.0	100	100	0.0	−1.0	111	43.9	62.3	58.4*
ARA‐2023	100	99.0	1.0	0.0	100	41.0	59.0	58.3*	103	5.9	97.0	94.9*
PET‐2023	103	96.3	3.8	2.8*	102	12.1	89.6	88.2*	100	77.0	22.97	22.1*
LIN‐2023	103	91.3	89.9	7.8*	103	17.5	84.9	83.1*	104	74.0	27.0	25.6*
TNG‐2023	103	88.1	12.3	11.1*	103	3.9	99.0	97.0*	114	8.8	104.0	96.8*
SUM‐2023	103	90.4	9.9	8.8*	103	63.0	38.1	36.7*	98	42.9	55.9	55.8*
BAR‐2023	103	85.4	15.1	13.9*	103	85.4	15.1	13.9*	103	46.1	55.5	54.0*
ANA‐2023	103	94.0	6.1	5.1*	103	19.9	82.5	80.7*	103	62.9	38.2	36.9*
PIE‐2023	103	85.3	15.1	13.9*	103	8.2	94.6	92.6*	111	5.0	105.4	99.5*
IRE‐2023	100	85.0	15.0	14.1*	100	10.0	90.0	89.4*	101	46.5	54.0	53.0*
PTY‐2023	102	82.5	17.8	16.7*	103	49.2	52.3	50.8*	100	22.0	78.0	77.4*
GOI‐2024	100	91.0	9.0	8.0*	100	0.0	100.0	99.5*	100	100.0	0.0	−1.0
PIE‐2024	100	93.0	7.0	6.0*	100	99.0	1.0	0.0	101	77.0	23.2	22.2*
AGU‐2024	103	89.7	10.6	9.4*	103	72.0	28.8	27.5*	103	72.0	28.8	27.5*
PTY‐2024	100	87.0	13.0	12.1*	102	40.7	60.5	59.2*	102	40.7	60.5	59.2*
SUM‐2024	103	85.4	15.1	13.9*	103	64.2	36.9	35.5*	103	78.7	21.9	20.7*
CNB‐2024	100	83.0	17.0	16.1*	100	0.0	100.0	99.5*	–	–	–	–
BAR‐2024	101	73.6	26.6	25.6*	101	0.0	101.0	100.0*	100	78.0	22.0	21.1*
IRE‐2024	100	85.6	14.4	13.5*	100	37.0	63.0	62.3*	100	64.4	35.6	34.7*
LIN‐2024	100	97.0	3.0	2.0*	100	95.0	5.0	4.1*	100	94.4	55.6	45.8*
JUA‐2024	100	68.4	31.6	30.7*	100	81.0	19.0	18.1*	100	61.0	39.0	38.2*
ARA‐2024	100	83.0	17.0	16.1*	100	68.0	32.0	31.1*	100	76.1	23.9	23.0*

^a^ Total number of insects bioassayed.

* Significant level *α* = 0.05 and critical value Zα = 1.645.

### 3.3 Cross‐resistance among chloride channel‐targeting insecticides

To evaluate potential cross‐resistance, the abamectin‐resistant GVT‐Aba population was tested with isocycloseram and fipronil. A substantial resistance ratio (RR = 1700.2) was confirmed for abamectin relative to the JDR1‐Sus strain. For fipronil, the RR was low (2.42‐fold) and statistically non‐significant. Isocycloseram showed a significant 9.6‐fold RR, which, although higher than those observed in field populations, may still reflect baseline variation (Table 5).

**Table 5:** Cross‐resistance pattern of abamectin – resistant *Phthorimaea absoluta* (GVT‐Aba) with other chloride channels acting insecticides

Population	Insecticide	*N* ^a^	Slope ± SE^b^	LC50 (CI95%)^c^	*χ* ^2^ (Prob., DF)^d^	RR_50_ (CI95%)^e^
JDR 1‐Sus	Abamectin (F145)	180	0.70 ± 0.14	0.014 (0.001–0.039)	8.48 (0.29, 7)	—
	Fipronil (F145)	270	1.85 ± 0.49	0.036 (0.008–0.064)	2.68 (0.84, 6)	—
	Isocycloseram (F122)	362	1.43 ± 0.12	0.0015 (0.0011–0.0019)	3.30 (0.77, 6)	—
GVT‐ Aba	Abamectin (F36)	240	1.32 ± 0.15	19.59 (11.33–33.26)	9.32 (0.15, 6)	1436.61 (459.94–4487.15)*
	Fipronil (F37)	240	1.53 ± 0.22	0.088 (0.051–0.132)	3.83 (0.69, 6)	2.42 (0.94–6.22)
	Isocycloseram (F36)	342	1.29 ± 0.16	0.0144 (0.00964–0.0214)	8.40 (0.31, 7)	9.58 (6.44–14.24)*

^a^ Total number of insects bioassayed.

^b^ Slope and standard error.

^c^ Milligrams of insecticide L^−1^ and 95% confidence interval.

^d^ Chi – square, probability, and degrees of freedom.

^e^ Resistance ratio: LC50 of each insecticide for GVT‐Sel compared to LC50 of the corresponding insecticide for JDR 1‐Sus by the lethal ratio test (Robertson *et al*. 2017).

* Resistance ratios were significant at 5% probability when the confidence limits of RR50 did not include the value 1.0.

### 3.4 Synergism

Enzymatic synergism bioassays indicated that metabolic detoxification plays a role in abamectin resistance. Co‐treatment with DEF, an esterase inhibitor, led to a 24.2‐fold increase in abamectin toxicity, highlighting the involvement of esterase‐based resistance. DEM (glutathione S‐transferase inhibitor) and PBO (cytochrome P450 inhibitor) also synergized abamectin (RS = 7.5 and 3.3, respectively), indicating roles for oxidative and conjugative metabolism. For isocycloseram, PBO produced the highest synergistic effect (RS = 6.6), implicating cytochrome P450s. DEM and DEF also increased toxicity (RS = 3.9 and 2.7, respectively), suggesting the involvement of GSTs and esterases. However, not all synergized resistance ratios were statistically significant, and baseline enzyme expression may explain the moderate effects observed for isocycloseram. In contrast, synergism ratios for abamectin were clearly significant, with synergized resistance ratios in the following order: PBO (123.5), DEM (82.6), and DEF (20.5) (Table 6).

**Table 6:** Synergism of abamectin and isocycloseram toxicity in both susceptible – (JDR1‐Sus) and abamectin selected – population (GVT‐Aba) of *Phthorimaea absoluta*

Population	Treatment	*N* ^a^	Slope ± SE^b^	LC50 (CI95%)^c^	*χ* ^2^ (DF, Prob.)^d^	SR_50_ (CI95%)^e^	synRR_50_ (CI95%)^f^
JDR1‐Sus	Abamectin (F147)	240	1.30 ± 0.21	0.062 (0.031–0.097)	1.35 (5, 0.92)	—	—
	Abamectin + PBO (F147)	210	1.24 ± 0.18	0.039 (0.024–0.058)	3.28 (5, 0.65)	1.04 (0.51–2.12)	—
	Abamectin + DEM (F147)	210	0.99 ± 0.17	0.027 (0.013–0.045)	2.91 (5, 0.71)	1.59 (0.80–3.17)	—
	Abamectin + DEF (F147)	210	1.24 ± 0.19	0.048 (0.019–0.089)	6.45 (5, 0.26)	1.28 (0.63–2.59)	—
	Isocycloseram (F147)	212	2.18 ± 0.28	0.009 (0.006–0.014)	5.00 (5, 0.41)	—	—
	Isocycloseram + PBO (F147)	210	2.06 ± 0.37	0.0019 (0.0011–0.002)	2.35 (5, 0.79)	5.13 (3.11–8.48)*	—
	Isocycloseram + DEM (F147)	210	1.74 ± 0.27	0.0044 (0.0026–0.0065)	1.47 (5, 0.91)	2.18 (1.28–3.70)*	—
	Isocycloseram + DEF (F147)	210	1.16 ± 0.19	0.0047 (0.00085–0.010)	10.28 (5, 0.06)	2.04 (1.13–3.67)*	—
GVT‐Aba	Abamectin (F38)	210	2.23 ± 0.36	24.06 (16.10–32.21)	2.34 (5, 0.80)	—	387.94 (206.88–727.47)*
	Abamectin + PBO (F38)	210	1.65 ± 0.25	7.29 (4.76–10.31)	1.12 (5, 0.95)	3.29 (2.00–5.42)*	123.47 (68.44–222.77)*
	Abamectin + DEM (F38)	210	1.24 ± 0.17	3.21 (1.67–5.26)	5.76 (5, 0.33)	7.48 (4.53–12.36)*	82.61 (46.49–146.77)*
	Abamectin + DEF (F38)	210	1.32 ± 0.23	0.98 (0.42–1.61)	0.92 (5, 0.97)	24.39 (12.11–49.13)*	20.46 (9.51–44.00)*
	Isocycloseram (F38)	210	1.61 ± 0.22	0.012 (0.0079–0.017)	2.29 (5, 0.80)	—	1.26 (0.77–2.07)
	Isocycloseram + PBO (F38)	210	1.65 ± 0.24	0.0018 (0.0011–0.0026)	4.17 (5, 0.52)	6.63 (3.77–11.65)*	0.98 (0.55–1.74)
	Isocycloseram + DEM (F38)	211	1.96 ± 0.26	0.0031 (0.0022–0.0042)	3.07 (5, 0.68)	3.93 (2.38–6.46)*	0.70 (0.41–1.20)
	Isocycloseram + DEF (F38)	210	1.41 ± 0.27	0.0044 (0.00043–0.010)	9.52 (5, 0.09)	2.68 (1.44–4.98)*	0.92 (0.42–1.98)

^a^ Total number of insects bioassayed.

^b^ Slope and standard error.

^c^ Milligrams of insecticide L^−1^ and 95% confidence interval.

^d^ Chi – square, probability, and degrees of freedom.

^e^ Synergism ratio: the LC50 of each insecticide without synergist compared to the LC50 of the corresponding insecticide with synergist, analyzed using the lethal ratio test (Robertson *et al*. 2017).

^f^ Synergized resistance ratio: the synergized LC50 of each insecticide for GVT‐Sel compared to synergized LC50 of the corresponding insecticide for JDR 1‐Sus by the lethal ratio test.

* Toxicity and synergism ratios were considered significant at a 5% probability level when the 95% confidence limits of RR50 and SR50 did not include value 1.0.

Piperonyl butoxide (PBO), diethyl maleate (DEM), and *S, S, S* – Tributyl phosphorotrithioate (DEF).

### 3.5 Correlation analysis

Pearson correlation analysis of LC₅₀ values and mortality data revealed significant positive correlations between fipronil and indoxacarb (r_p_ = 0.807, *P* = 0.00048, *n* = 14), and between tolfenpyrad and isocycloseram (r_p_ = 0.732, *P* = 0.0029, *n* = 14), following false discovery rate (FDR) correction. No other correlations were statistically significant.

## 4 DISCUSSION

Understanding why resistance emerges so rapidly after the introduction of new insecticides is central to sustainable pest management. Each new active ingredient enters the market with the promise of improved control and resistance mitigation. Yet history shows a recurring pattern: optimism fades as pest populations adapt, often within just a few years. Intensive, uncoordinated applications amplify selection pressure, favoring resistant genotypes and shortening product lifespan—especially where integrated resistance management (IRM) programs are weak or absent.^30^, ^31^ Baseline susceptibility studies address this challenge by revealing the natural range of insecticide sensitivity before widespread field exposure. These data provide an empirical foundation for resistance monitoring, help define diagnostic concentrations, and inform early IRM strategies.^32^, ^33^

Establishing reliable baselines allows early detection of changes before control failures occur. Here, susceptibility to two recently introduced insecticides—isocycloseram and tolfenpyrad—was characterized across *P. absoluta* populations from Brazil. These compounds represent new modes of action, expanding the scope of current monitoring programs. Resistance management begins with understanding why susceptibility baselines matter: they provide an early warning system, revealing subtle shifts in population response before control failures occur. This is particularly urgent for *P. absoluta*, a species with a well‐documented history of rapidly evolving resistance. Baseline assays confirmed high susceptibility of *P. absoluta* populations to isocycloseram, and tolfenpyrad. Introduced for tomato pest control in 2020, tolfenpyrad produced consistent LC₅₀ values across populations but variable LC₉₉ estimates. Such variability at high concentrations suggests heterogeneous tolerance—an early signal of potential resistance evolution. Isocycloseram displayed remarkable efficacy, with LC_99_ values below 1 mg L^−1^ in nearly all populations. Yet tolerance ratios up to 30‐fold were detected, particularly in a strain previously selected with abamectin (GVT‐Aba), suggesting metabolic cross‐resistance. Accordingly, diagnostic concentrations of 0.3 mg L^−1^ for isocycloseram and 47 mg L^−1^ for tolfenpyrad were defined based in LC₉₉ estimated from pooled laboratory and field populations.^34^, ^35^ Rather than anchoring the decision solely on a single population or on prior assumptions of susceptibility uniformity, the pooled estimate was used to capture a broader and more representative range of upper‐limit responses. This strategy minimizes overreliance on outlier or idealized laboratory data and enhances the robustness of diagnostic doses as practical surveillance tools—sensitive enough to detect early resistance while remaining representative of field variability.^36^, ^37^

Surveys conducted in 2023–2024 also provided comparative data for older insecticides. Resistance frequencies varied among populations: abamectin showed generally lower levels, indoxacarb intermediate, and fipronil consistently high. The persistence of fipronil resistance, despite its unregistered *status* for *P. absoluta* in Brazil, suggests off‐label or indirect exposure, possibly through environmental contamination or crop rotation practices. Moreover, the absence of correlation between abamectin and fipronil resistance indicates that these patterns are independent, reinforcing the lack of cross‐resistance between both compounds. Together, these results underscore the value of integrating new active ingredients into resistance surveillance and highlight the need for continued monitoring to anticipate shifts before they become operationally significant.

Although no resistance to fipronil has been documented in *P. absoluta* to date, several populations have evolved resistance to abamectin^6^, ^38^ and indoxacarb.^21^, ^39^ Mechanistically, these compounds act through distinct neural targets: abamectin activates glutamate‐ and GABA‐gated chloride channels, inducing paralysis by uncontrolled chloride influx, fipronil, a phenylpyrazole, antagonizes GABA receptors (RDL‐type), blocking chloride entry and causing hyperexcitation. Despite these differences, significant cross‐resistance was detected between fipronil and indoxacarb, and between tolfenpyrad and isocycloseram—patterns that likely reflect shared metabolic detoxification rather than target‐site overlap.

Interestingly, a population highly resistant to chlorantraniliprole remained fully susceptible to isocycloseram—evidence that distinct detoxification pathways are involved. By 2024, slight tolerance shifts became apparent in newly collected populations, hinting that selection pressure may already be underway following isocycloseram's commercial debut. This emphasizes the need for proactive resistance management—before efficacy losses become measurable in the field. As argued by Brent (1986)^16^ and Roush & Miller (1986),^26^ early detection and dynamic adjustment of monitoring protocols remain central to sustaining insecticide effectiveness and prolonging the lifespan of novel chemistries. These early tolerance patterns also raise mechanistic questions about whether isocycloseram shares detoxification routes with other modern insecticides—a possibility that could shape cross‐resistance outcomes and monitoring strategies.

Previous work demonstrated metabolic cross‐resistance between fipronil and indoxacarb in *P. xylostella* after selection with fipronil.^40^ Although no such reports exist for isocycloseram or tolfenpyrad, their shared structural feature—a central amide moiety—raises the possibility of common metabolic routes mediated by amidases or monooxygenases. Supporting this, Lee *et al*. (2024)^41^ found that piperonyl butoxide (PBO) enhanced isocycloseram toxicity in *Blattella germanica*, implicating cytochrome P450 monooxygenases. Similar P450‐driven detoxification has been described for tebufenpyrad, another pyrazole acaricide,^42^ and P450 inhibition has been linked to restored tolfenpyrad susceptibility in pyrethroid‐resistant mosquitoes.^10^ However, the strong positive association between isocycloseram and tolfenpyrad mortality may simply reflect the overall susceptibility of the populations tested—most exhibited near‐total mortality to both compounds. In such cases, apparent correlations can emerge from uniformly high toxicities rather than shared resistance mechanisms. Clarifying whether metabolism indeed links these two molecules will require targeted synergism bioassays and molecular analyses.

The synergism assays further explored resistance mechanisms. The GVT‐Aba population showed a ~10‐fold tolerance to isocycloseram but not to fipronil, ruling out shared target‐site resistance. Enzyme inhibitor assays revealed significant synergism with isocycloseram in the susceptible population—particularly for monooxygenase inhibitors—while abamectin synergism was more strongly associated with esterase inhibitors. This contrast suggests distinct metabolic pathways: monooxygenases predominantly mediating isocycloseram detoxification, while esterases play a larger role in abamectin metabolism. The elevated tolerance to isocycloseram in the abamectin‐selected population may reflect a broader metabolic capacity, possibly *via* upregulated oxidases. Although esterases and GSTs cannot be entirely excluded from contributing to isocycloseram metabolism, the data emphasizes the importance of oxidases in its detoxification. Given the increasing adoption of both isocycloseram and abamectin in Brazilian tomato production, these results reinforce the necessity for strategic deployment and resistance monitoring to preserve their long‐term efficacy.

In conclusion, our study establishes critical baseline susceptibility data and resistance monitoring tools for *P. absoluta* populations in Brazil, targeting novel (isocycloseram, tolfenpyrad) and old‐fashioned (abamectin, indoxacarb, fipronil) insecticides. Diagnostic doses were proposed, allowing early detection of resistance. Results indicate that detoxification enzymes—especially esterases and oxidases—play a central role in resistance to abamectin, while isocycloseram remains largely unaffected by metabolic cross‐resistance. Positive correlations between some compounds emphasize the need for careful rotation strategies considering both MoA and shared metabolic pathways. The insights presented here provide robust scientific and operational guidance for resistance monitoring and sustainable chemical control of *P. absoluta* in Brazil and elsewhere. Continued monitoring, integration with non‐chemical control, and grower education are essential for preserving the efficacy of these critical insecticides.

## CONFLICT OF INTEREST

The authors declare no conflicts of interest.

## AUTHOR CONTRIBUTIONS

VQ and HAAS conceived research. VQ, MO, JVCRD and TPL conducted the experiments. HAAS contributed material. VQ, TPL and HAAS analyzed the data and conducted statistical analyses. HAAS secured funding. VQ data curation. VQ, HAAS, and TPL wrote the manuscript. All authors read, contributed to, and approved the manuscript.

## Supporting information


**Figure S1.** Concentration–mortality curves for isocycloseram. The vertical line indicates the diagnostic concentration of 0.3 mg L⁻^1^. The pattern area represents the label‐recommended dose range for the control of *P. absoluta* in tomato. The blue dashed curve corresponds to the fitted values obtained using the pooled dataset.
**Figure S2.** Concentration–mortality curves for tolfenpyrad. The vertical line indicates the diagnostic concentration of 47 mg L⁻^1^. The pattern area represents the label‐recommended dose range for the control of *P. absoluta* in tomato. The blue line corresponds to the fitted values obtained using the pooled dataset.

## Data Availability

The data that support the findings of this study are available from the corresponding author upon reasonable request.

## References

[1] Desneux N , Luna MG , Guillemaud T and Urbaneja A , The invasive south American tomato pinworm, Tuta absoluta, continues to spread in afro‐Eurasia and beyond: the new threat to tomato world production. J Pest Sci 84:403–408 (2011).

[2] Guedes RNC and Siqueira HAA , The tomato borer *Tuta absoluta*: insecticide resistance and control failure. CABI Rev 2012:1–7 (2012).

[3] Biondi A , Guedes RNC , Wan F‐H and Desneux N , Ecology, worldwide spread, and Management of the Invasive South American Tomato Pinworm, *Tuta absoluta*: past, present, and future. Annu Rev Entomol 63:239–258 (2018). 28977774 10.1146/annurev-ento-031616-034933

[4] Guedes RNC , Roditakis E , Campos MR , Haddi K , Bielza P , Siqueira HAA *et al*., Insecticide resistance in the tomato pinworm *Tuta absoluta*: patterns, spread, mechanisms, management and outlook. J Pest Sci 92:1329–1342 (2019).

[5] Silva TBM , Silva WM , Campos MR , Silva JE , Ribeiro LMS and Siqueira HAA , Susceptibility levels of Tuta absoluta (Meyrick) (Lepidoptera: Gelechiidae) to minor classes of insecticides in Brazil. Crop Prot 79:80–86 (2016).

[6] Quintela V , Pereira DL , Langa TP , de Oliveira M and de Siqueira HÁA , Field‐evolved resistance in *Phthorimaea absoluta* to abamectin: genetic foundations, female‐linked traits, and cross‐resistance pattern. Pest Manag Sci 81:3867–3877 (2025). 40050030 10.1002/ps.8753

[7] Silva GA , Picanço MC , Bacci L , Crespo ALB , Rosado JF and Guedes RNC , Control failure likelihood and spatial dependence of insecticide resistance in the tomato pinworm, Tuta absoluta. Pest Manag Sci 67:913–920 (2011). 21394881 10.1002/ps.2131

[8] Campos MR , Silva TB , Silva WM , Silva JE and Siqueira HA , Susceptibility of *Tuta absoluta* (Lepidoptera: Gelechiidae) Brazilian populations to ryanodine receptor modulators: baseline of susceptibility of *Tuta absoluta* to diamides. Pest Manag Sci 71:537–544 (2015). 24863675 10.1002/ps.3835

[9] Silva JE , Assis CPO , Ribeiro LMS and Siqueira HAA , Field‐evolved resistance and cross‐resistance of Brazilian *Tuta absoluta* (Lepidoptera: Gelechiidae) populations to Diamide insecticides. J Econ Entomol 109:2190–2195 (2016). 27427509 10.1093/jee/tow161

[10] Lees RS , Ismail HM , Logan RAE , Malone D , Davies R , Anthousi A *et al*., New insecticide screening platforms indicate that mitochondrial complex I inhibitors are susceptible to cross‐resistance by mosquito P450s that metabolize pyrethroids. Sci Rep 10:16232 (2020). 33004954 10.1038/s41598-020-73267-x PMC7530702

[11] Blythe J , Earley FGP , Piekarska‐Hack K , Firth L , Bristow J , Hirst EA *et al*., The mode of action of isocycloseram: A novel isoxazoline insecticide. Pestic Biochem Physiol 187:105217 (2022). 36127059 10.1016/j.pestbp.2022.105217

[12] Arena JP , Liu KK , Paress PS , Frazier EG , Cully DF , Mrozik H *et al*., The mechanism of action of avermectins in Caenorhabditis elegans: correlation between activation of glutamate‐sensitive chloride current, membrane binding, and biological activity. J Parasitol 81:286–294 (1995). 7707209

[13] Jansson RK and Dybas RA , Avermectins: Biochemical Mode of Action, Biological Activity and Agricultural Importance, Insecticides with Novel Modes of Action, Berlin (1998).

[14] Silver KS , Song W , Nomura Y , Salgado VL and Dong K , Mechanism of action of sodium channel blocker insecticides (SCBIs) on insect sodium channels. Pestic Biochem Physiol 97:87–92 (2010). 24013950 10.1016/j.pestbp.2009.09.001 PMC3765036

[15] Zhao X , Yeh JZ , Salgado VL and Narahashi T , Sulfone metabolite of Fipronil blocks γ‐aminobutyric acid‐ and glutamate‐activated chloride channels in mammalian and insect neurons. J Pharmacol Exp Ther 314:363–373 (2005). 15701711 10.1124/jpet.104.077891

[16] Pesticide Resistance: Strategies and Tactics for Management, Vol. 619. National Academies Press, Washington, D.C. (1986).

[17] Insecticide Resistance Action Committee|IRAC , Insecticide Resistance Action Committee, 14 April 2025 https://irac-online.org/ [accessed 19 September 2025].

[18] Pereira A E , Souza D , Zukoff SN , Meinke LJ and Siegfried BD , Cross‐resistance and synergism bioassays suggest multiple mechanisms of pyrethroid resistance in western corn rootworm populations Guedes RNC. PLoS One 12:e0179311 (2017). 28628635 10.1371/journal.pone.0179311 PMC5476265

[19] IRAC I, IRAC Susceptibility Test Method 022, IRAC Insecticide Resistance Action Committee, 26 August 2023 https://irac-online.org/methods/tuta-absoluta-larvae/ [accessed 26 August 2023].

[20] Kumar S , Sharma AK , Nagar G and Ghosh S , Determination and establishment of discriminating concentrations of malathion, coumaphos, fenvalerate and fipronil for monitoring acaricide resistance in ticks infesting animals. Ticks Tick‐borne Dis 6:383–387 (2015). 25840925 10.1016/j.ttbdis.2015.03.003

[21] Roditakis E , Mavridis K , Riga M , Vasakis E , Morou E , Rison JL *et al*., Identification and detection of indoxacarb resistance mutations in the *para* sodium channel of the tomato leafminer, *Tuta absoluta*: Indoxacarb resistance mechanisms in *Tuta absoluta* . Pest Manag Sci 73:1679–1688 (2017). 28019074 10.1002/ps.4513

[22] Abbott WS , A method of computing the effectiveness of an insecticide. J Econ Entomol 18:265–267 (1925).

[23] Robertson JL , Jones MM , Olguin E and Alberts , Bioassays with arthropods, 3rd edn. CRC Press, Taylor & Francis Group, Boca Raton (2017).

[24] Snedecor GW and Cochran W , Statistical Methods, 8th edn. Iowa State University Press, Ames, IA (1986).

[25] R Core Team , Vienna, AustriaR (2025).

[26] Roush RT and Miller GL , Considerations for Design of Insecticide Resistance Monitoring Programs. J Econ Entomol 79:293–298 (1986).

[27] Wei T and Simko V , corrplot: Visualization of a Correlation Matrix, 0.95 (2025).

[28] Benjamini Y and Hochberg Y , Controlling the false discovery rate. J R Stat Soc: Ser B 57:289–300 (1995).

[29] Guedes RNC , Insecticide resistance, control failure likelihood and the first law of geography: insecticide resistance and control failure likelihood. Pest Manag Sci 73:479–484 (2017). 27714951 10.1002/ps.4452

[30] Bass C , Denholm I , Williamson MS and Nauen R , The global status of insect resistance to neonicotinoid insecticides. Pestic Biochem Physiol 121:78–87 (2015). 26047114 10.1016/j.pestbp.2015.04.004

[31] Hawkins NJ , Bass C , Dixon A and Neve P , The evolutionary origins of pesticide resistance: the evolutionary origins of pesticide resistance. Biol Rev 94:135–155 (2019). 29971903 10.1111/brv.12440 PMC6378405

[32] Benvenuto EA d S , de Araujo EL , Ribeiro LM d S , da Silva KWL , Pereira DL , de Oliveira M *et al*., Bioassay tray for assessing susceptibility of Liriomyza sativae Blanchard (Diptera: Agromyzidae) to reduced‐risk insecticides and resistance monitoring in Brazil. Phytoparasitica 52:80 (2024).

[33] Marçon PCRG , Young LJ , Steffey KL and Siegfried BD , Baseline susceptibility of European corn borer (Lepidoptera: Crambidae) to *bacillus thuringiensis* toxins. J Econ Entom 92:279–285 (1999).

[34] Fardisi M , Gondhalekar AD and Scharf ME , Development of diagnostic insecticide concentrations and assessment of insecticide susceptibility in German cockroach (Dictyoptera: Blattellidae) field strains collected from public housing. J Econ Entom 110:1210–1217 (2017). 28334270 10.1093/jee/tox076 PMC5444675

[35] Bosch D , Rodríguez MA , Depalo L and Avilla J , Determination of the baseline susceptibility of European populations of *Cydia pomonella* (Lepidoptera: Tortricidae) to Chlorantraniliprole and the role of cytochrome P450 monooxygenases. J Econ Entom 111:844–852 (2018). 29438567 10.1093/jee/toy020

[36] ffrench‐Constant RH and Roush RT , Resistance Detec tion and documentation: the relative roles of Pesticidal and biochemical assays, in Pesticide Resistance in Arthropods, ed. by Roush RT and Tabashnik BE . Springer US, Boston, MA, pp. 4–38 (1990).

[37] Azizi M and Khajehali J , Evaluation of resistance to Abamectin in the populations of Tuta absoluta. J Agri Sci Technol 24:379–391 (2022).

[38] Aboutalebian‐Soureshjani A , Rafiee‐Dastjerdi H , Naseri B , Hassanpour M and Khajehali J , Indoxacarb resistance in Iranian populations of *Tuta absoluta* (Lepidoptera: Gelechiidae): cross‐resistance, biochemical and molecular mechanisms. Pestic Biochem Physiol 196:105633 (2023). 37945235 10.1016/j.pestbp.2023.105633

[39] Halliday RW and Burnhaw KP , Choosing the optimal diagnostic dose for monitoring insecticide resistance. J Econ Entomol 83:1151–1159 (1990).

[40] Sayyed AH and Wright DJ , Fipronil resistance in the diamondback moth (Lepidoptera: Plutellidae): inheritance and number of genes involved. J Econ Entomol 97:2043–2050 (2004). 15666763 10.1093/jee/97.6.2043

[41] Lee S‐H , So J , Kund GS , Lum J‐Y , Trinh E , Ta EL *et al*., Toxicity of isocycloseram, an isoxazoline insecticide, against laboratory and field‐collected German cockroaches (Blattodea: Ectobiidae), ed. by Appel, A. J Econ Entomol 117:1086–1094 (2024). 38624063 10.1093/jee/toae079

[42] Pottelberge SV , Leeuwen TV , Nauen R and Tirry L , Resistance mechanisms to mitochondrial electron transport inhibitors in a field‐collected strain of *Tetranychus urticae* Koch (Acari: Tetranychidae). Bull Entomol Res 99:23–31 (2009). 18590597 10.1017/S0007485308006081

