# Supplementary material for: Susceptibility of Phthorimaea absoluta (Meyrick) (Lepidoptera: Gelechiidae) to novel and established insecticides in Brazil: resistance survey, baseline, and implications for management
Source: Pest Manag Sci. 2026 Feb 22;82(6):5662–71. doi: 10.1002/ps.70669 (PMC13158445; doi:10.1002/ps.70669)
Supplement: Supplementary file 1 — Figure S1. Concentration–mortality curves for isocycloseram. The vertical line indicates the diagnostic concentration of 0.3 mg L⁻1. The pattern area represents the label‐recommended dose range for the control of P. absoluta in tomato. The blue dashed curve corresponds to the fitted values obtained using the pooled dataset. Figure S2. Concentration–mortality curves for tolfenpyrad. The vertical line indicates the diagnostic concentration of 47 mg L⁻1. The pattern area represents the label‐recommended dose range for the control of P. absoluta in tomato. The blue line corresponds to the fitted values obtained using the pooled dataset. [file PS-82-5662-s001.docx]

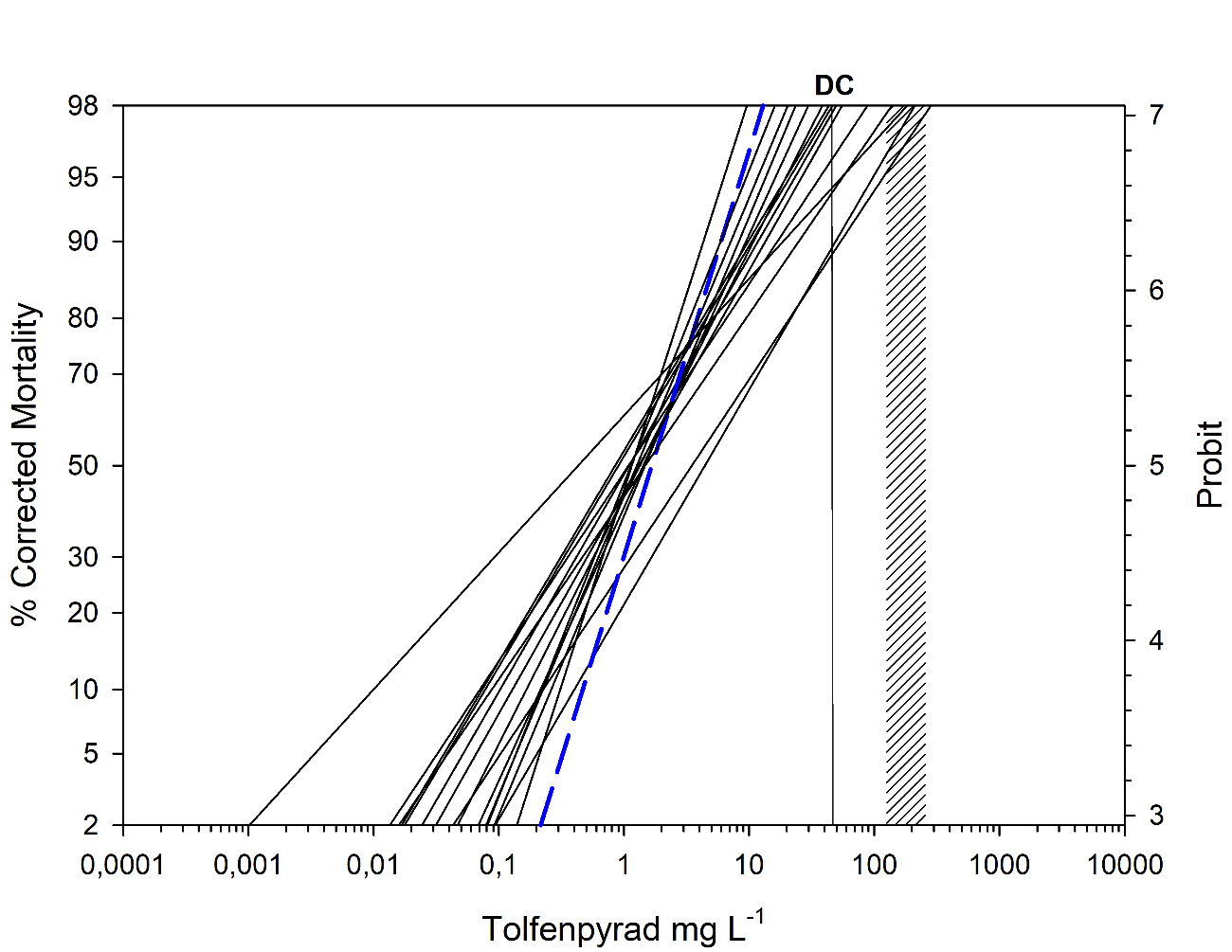


Fig. Sup. 1. Concentration–mortality curves for isocycloseram. The vertical line indicates the diagnostic concentration of 0.3 mg L⁻¹. The pattern area represents the label-recommended dose range for the control of *P. absoluta* in tomato. The blue dashed curve corresponds to the fitted values obtained using the pooled dataset.


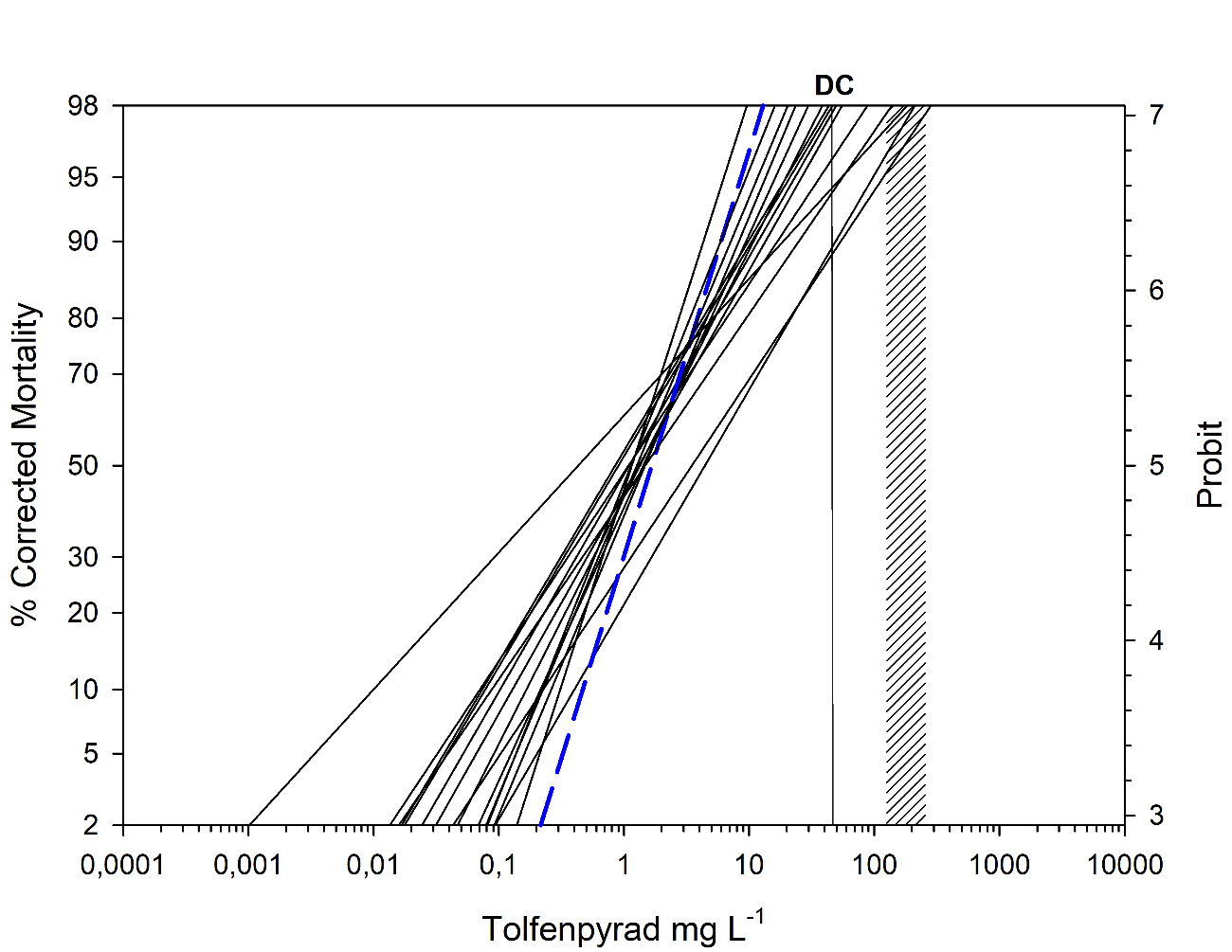


Fig. Sup. 2. Concentration–mortality curves for tolfenpyrad. The vertical line indicates the diagnostic concentration of 47 mg L⁻¹. The pattern area represents the label-recommended dose range for the control of *P. absoluta* in tomato. The blue line corresponds to the fitted values obtained using the pooled dataset.
